# A Case of Bronchial Foreign Body Aspiration Caused by Inhaling a Mukago (Edible Yam Bulbil) Presenting With Persistent Cough

**DOI:** 10.1002/rcr2.70346

**Published:** 2025-09-18

**Authors:** Takayuki Yamamoto, Yukihiro Sugimoto, Yu Tsukasa, Ririko Shinozaki, Daiki Hasama, Ryota Aoki, Hirofumi Nakano, Sosei Abe, Masanori Takayama

**Affiliations:** ^1^ Department of Respiratory Medicine Fukuoka Seisyukai Hospital Fukuoka Japan; ^2^ Department of Thoracic Surgery Fukuoka Seisyukai Hospital Fukuoka Japan

**Keywords:** aspiration, bronchoscopy, foreign body, persistent cough, yam bulbil

## Abstract

An 85‐year‐old man presented with a persistent cough accompanied by sputum production. He was initially treated with antibiotics and expectorants but showed no improvement. Chest computed tomography revealed a dense nodule in the right bronchus intermedius. On detailed history‐taking, he recalled eating mukago—a small edible bulbil of *Dioscorea japonica* that is occasionally consumed in Japan—shortly before symptom onset. Flexible bronchoscopy identified an oval, hard foreign body lodged in the bronchus, which was successfully removed using biopsy forceps. The extracted object was confirmed to be a mukago. His cough disappeared after removal. Our case highlights the importance of considering foreign body aspiration in elderly patients presenting with unexplained chronic cough, even in the absence of overt choking episodes. Thorough dietary history‐taking, appropriate imaging, and bronchoscopy are essential for timely diagnosis and effective management of foreign body aspiration.

## Graphical abstract

This case report describes an elderly patient presenting with a persistent cough due to the aspiration of a mukago (edible yam bulbil), which is occasionally eaten in Japan. The foreign body was confirmed by chest CT and removed by flexible bronchoscopy. Clinicians should consider the possibility of airway foreign body aspiration in elderly patients with chronic respiratory symptoms, even in the absence of a clear history of food aspiration.

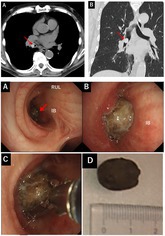

## 1 Introduction

Airway foreign bodies seen in adults can include various materials, such as food particles and dental prostheses [1]. In elderly individuals, aspiration events may remain unrecognised and are often identified incidentally during the evaluation of chronic cough, haemoptysis, or recurrent pneumonia [2]. Its diagnosis can be delayed, particularly when the foreign body is radiolucent or when there is no clear history of aspiration [3]. We report a case of airway foreign body aspiration caused by accidental inhalation of a yam bulbil (mukago), which initially presented as prolonged cough. The present case underscores the importance of maintaining a high index of suspicion for airway foreign body aspiration in older adults and highlights the role of careful dietary history‐taking. A brief literature review is also provided.

## 2 Case Report

An 85‐year‐old man developed cough and sputum production beginning in early September. His medical history included type 2 diabetes mellitus, hypertension, non‐valvular atrial fibrillation, benign prostatic hyperplasia, and prior cerebral infarction. He had no smoking history and had worked as a civil servant. His medications included edoxaban, nifedipine, olmesartan, naftopidil, teneligliptin, insulin glulisine, insulin glargine, and probiotics.

He was initially treated with expectorants and antibiotics at a local clinic, but he showed no improvement. In October, he underwent chest computed tomography (CT) at another clinic, revealing a high‐density nodule in the right intermediate bronchus. He was referred to our department for further evaluation.

**FIGURE 1:**
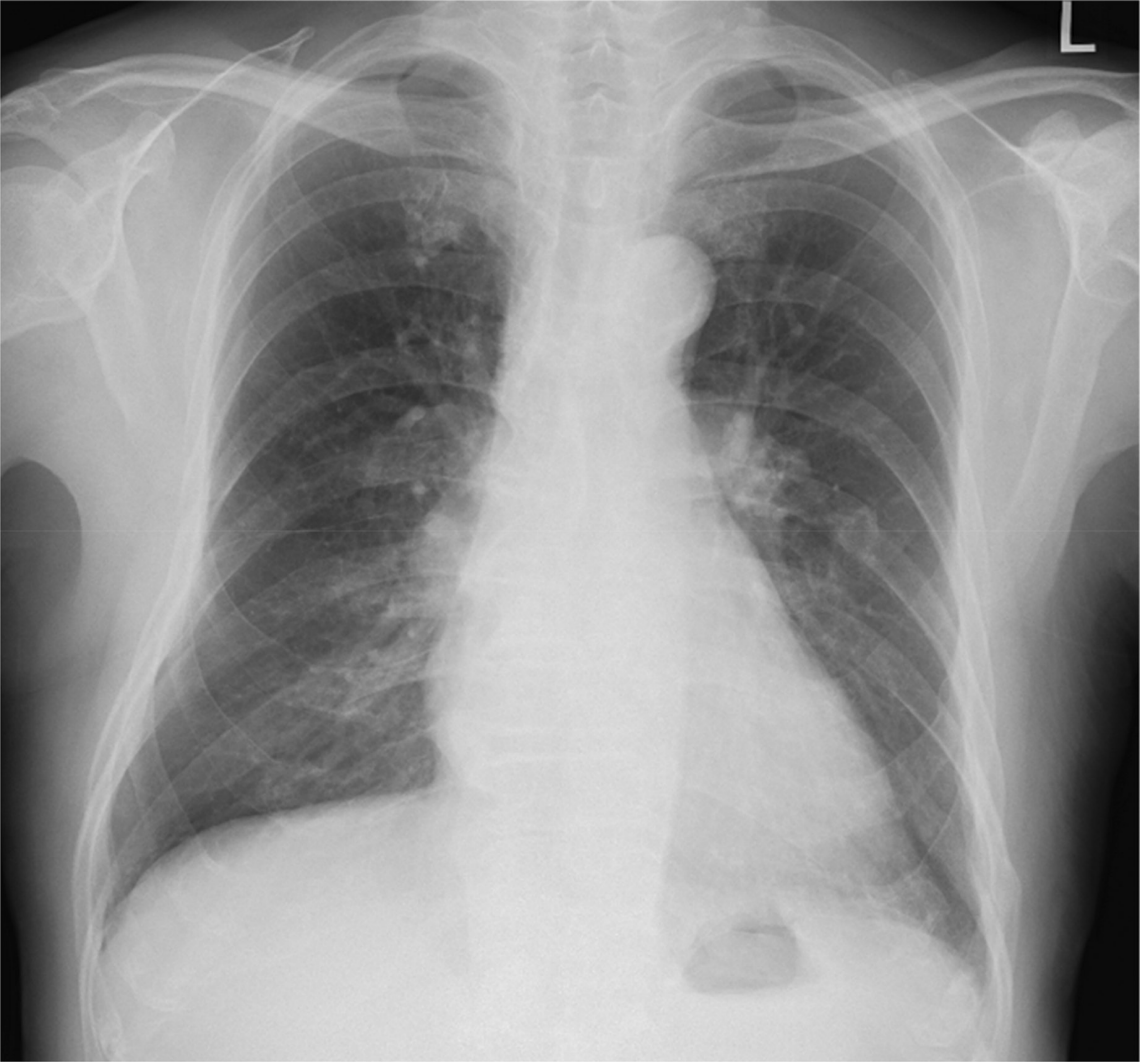
A chest radiograph showing no obvious abnormalities.

**FIGURE 2:**
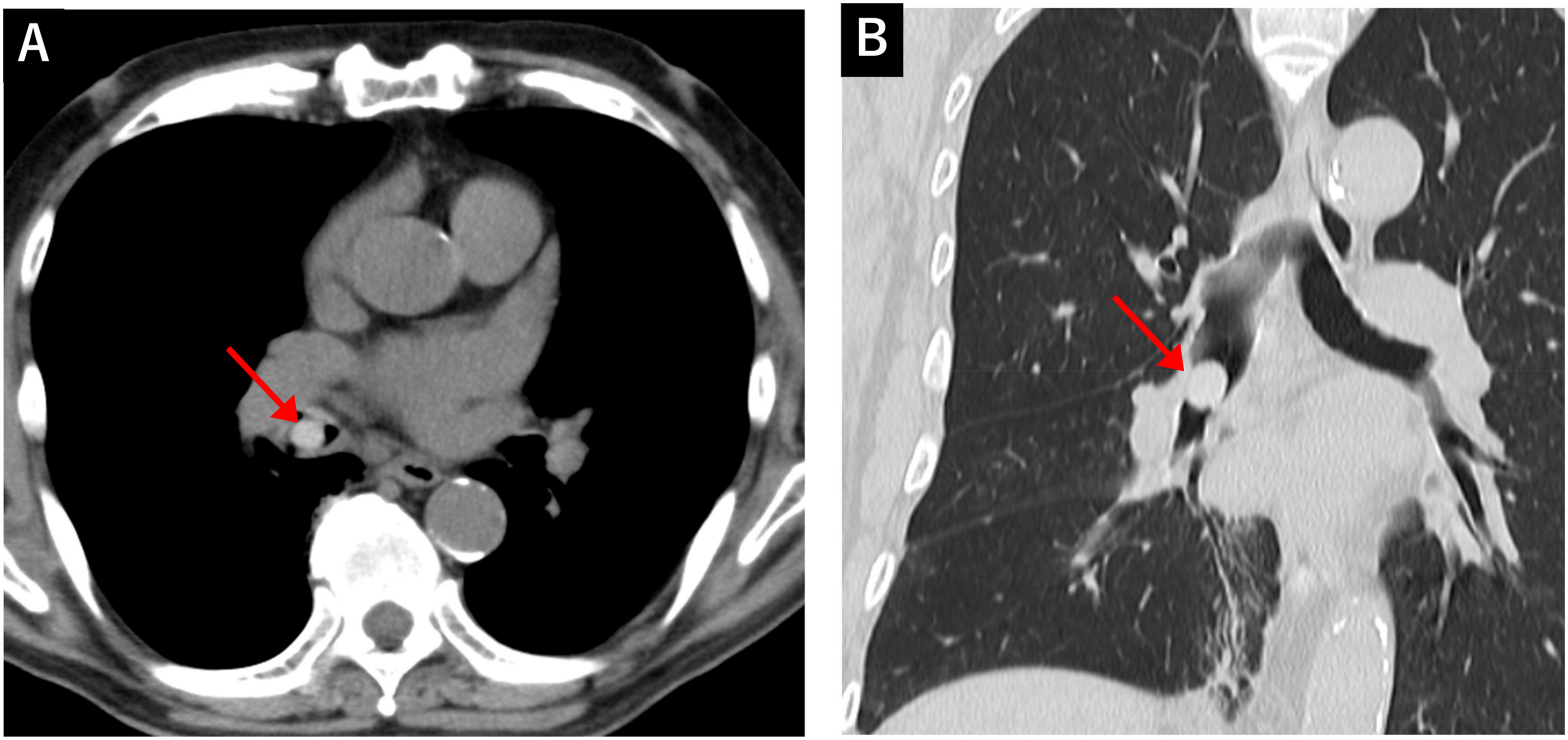
A non‐contrast‐enhanced chest computed tomography scan demonstrating a high‐density nodule in the right bronchus intermedius (indicated by red arrows). (A) Axial view with mediastinal window setting. (B) Coronal view with lung window setting.

On presentation, he was afebrile and had stable vital signs. Physical examination demonstrated unremarkable findings. Laboratory testing showed mild leucocytosis (8994/μL) with neutrophil predominance and a slightly elevated C‐reactive protein level (0.62 mg/dL). Chest radiography (Figure 1) revealed no clear abnormalities. Chest CT (Figure 2) demonstrated a high‐density lesion obstructing the right intermediate bronchus.

**FIGURE 3:**
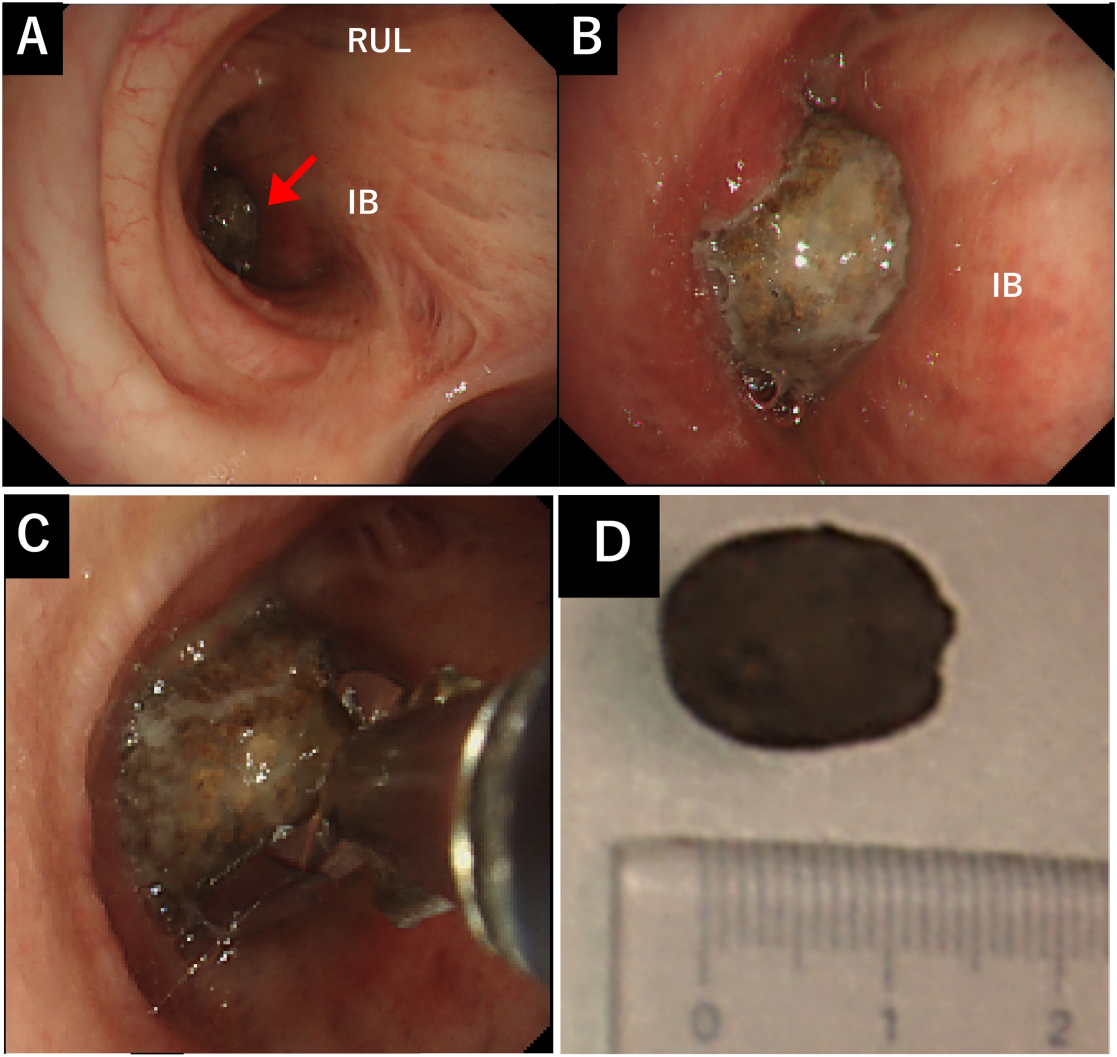
(A–C) Bronchoscopy showing an oval‐shaped foreign body (mukago, red arrow) obstructing the entrance to the right intermediate bronchus. The right upper lobe bronchus is also indicated for anatomical reference. (IB, intermediate bronchus; RUL, right upper lobe bronchus). (D) The retrieved mukago.

**FIGURE 4:**
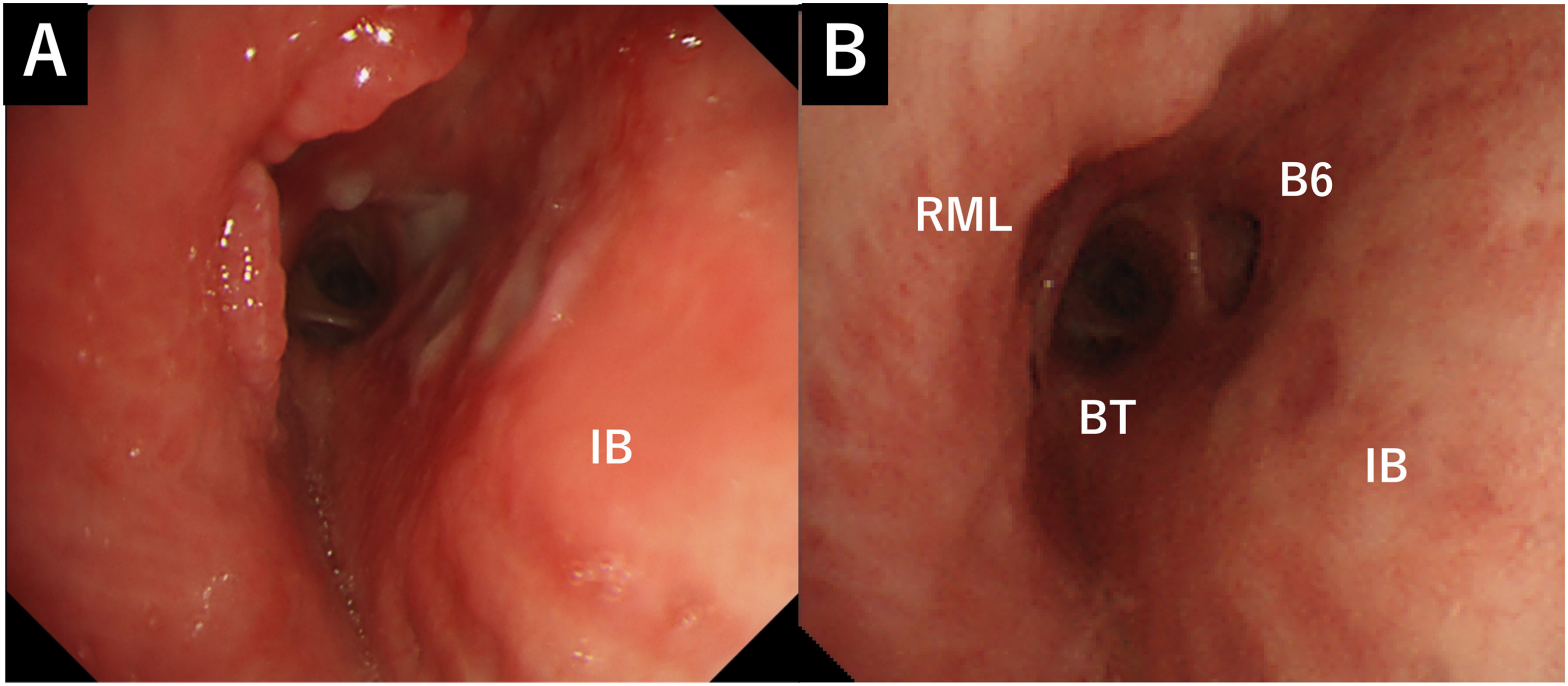
(A) Surrounding airway mucosa exhibiting erythema, ulceration and granulation tissue. (B) At 1 month after the foreign body removal, the bronchial lumen showed a marked improvement. (B6, superior segmental bronchus of the lower lobe; BT, basal trunk; RML, right middle lobe bronchus).

Flexible bronchoscopy revealed an oval foreign body lodged in the right bronchus intermedius (Figure 3A,B). Under sedation with midazolam, an endotracheal tube(8.0 mm internal diameter) was inserted, and a flexible bronchoscope (Olympus IT260, outer diameter 6.0 mm, 2.8 mm working channel) was advanced. Alligator forceps (Olympus FG‐49L‐1, maximum outer diameter 2.65 mm) were used to retrieve the foreign body (Figure 3C), which was elastic‐hard and measured approximately 1.3 × 0.8 cm. To prevent dislodgement during extraction, the foreign body was removed together with the forceps and bronchoscope as a single unit. Macroscopic examination identified it as a desiccated edible bulbil (mukago) (Figure 3D). The surrounding bronchial mucosa showed erythema, ulceration and granulation (Figure 4A). Follow‐up bronchoscopy after 1 month revealed marked resolution of the mucosal inflammation observed at the initial examination (Figure 4B). After removal, the patient's cough resolved.

## 3 Discussion

Airway foreign bodies can be organic such as legumes, fish bones and vegetables, or inorganic such as dentures, pins and coins [4]. A bimodal age distribution has been reported among cases, with the incidence peaking in children aged < 3 years and adults aged > 70 years [5, 6]. In children, legumes, including peanuts, are the most common type of foreign body, but in adults, non‐food items such as dentures are more common [7]. The aspiration of legumes or similar food materials is rare and may depend on regional dietary habits [1].

In the present case, the absence of a clear aspiration episode contributed to a diagnostic delay. Chronic cough in adults can have numerous aetiologies, including asthma, post‐infectious cough, gastroesophageal reflux disease and atopic cough. As airway foreign body aspiration can mimic these conditions, clinicians should consider this possibility, particularly in elderly patients with unexplained symptoms. Altogether, 45%–88% of adults are initially misdiagnosed as having pneumonia or a malignancy [8, 9].

A detailed dietary history taking was essential in our case. Through systematic questioning about food intake, mukago consumption was identified as the likely source. Mukago is an aerial bulbil of the yam plant ( *Dioscorea japonica* ), which serves as a vegetative reproductive organ that falls to the ground and grows into a new plant [10]. In Japan, mukago is a seasonal food commonly boiled or cooked with rice in some regions, including Hokkaido, Aomori and Okinawa. Although it is edible, its size and shape can predispose to aspiration, particularly in the elderly with decreased protective reflexes.

Radiolucent foreign bodies, including legumes, are difficult to identify on plain radiographs, underscoring the importance of CT imaging for detection. When a fat‐density lesion is visualised within the airway, differential diagnoses include lipoma and hamartoma in addition to aspirated material [11]. Flexible bronchoscopy is generally used for diagnosis and removal, with a reported success rate exceeding 90% [7]. Various retrieval devices—standard forceps, baskets and snares—should be selected according to the object's characteristics.

In this case, the foreign body was successfully retrieved by flexible bronchoscopy under sedation; however, in some situations, removal can be complicated by granulation tissue or bleeding risk, requiring collaboration with thoracic surgeons and readiness to convert to rigid bronchoscopy or surgical extraction. Moreover, potential complications such as acute airway obstruction due to dislodgement into the larynx, hypoxemia, bleeding, and airway perforation leading to pneumothorax or mediastinal emphysema must also be considered. Therefore, in our patient, endotracheal intubation was performed, and the thoracic surgery team was informed in advance to prepare for possible massive bleeding or other complications.

Beyond this single case, with the ongoing population ageing, the number of challenging cases of foreign body aspiration is expected to increase. Therefore, it is essential to conduct thorough history‐taking that includes patients' dietary habits, preferences, and food culture, and for each institution to carefully consider appropriate diagnostic approaches and strategies for safe removal of airway foreign bodies.

In conclusion, we report a case of airway foreign body aspiration caused by mukago inhalation, which presented as a prolonged cough. For elderly patients with or without specific respiratory symptoms or abnormal chest radiographic findings, clinicians should consider the possibility of airway foreign body aspiration and perform thorough history‐taking.

## Author Contributions

Drs. Takayuki Yamamoto and Yukihiro Sugimoto were responsible for the conception and design of the report and drafted the manuscript. Drs. Yu Tsukasa, Ririko Shinozaki, Daiki Hasama, Ryota Aoki, Sosei Abe, Hirofumi Nakano, and Masanori Takayama performed the bronchoscopic procedure and supervised the manuscript preparation. All authors read and approved the final manuscript and agree to be accountable for all aspects of the work.

## Consent

The authors declare that written informed consent was obtained for the publication of this manuscript and accompanying images using the consent form provided by the Journal.

## Conflicts of Interest

The authors declare no conflicts of interest.

## Data Availability

Data sharing not applicable to this article as no datasets were generated or analysed during the current study.
